# Raising patient voices in medical education: an assessment of patient perceived effect of social determinants of health conversations and the patient-physician relationship on quality of obstetric care, to inform the development of patient driven medical education curricula

**DOI:** 10.3389/frph.2024.1283390

**Published:** 2024-02-15

**Authors:** S. Brito, A. Rau, C. Escobar, P. Garza, I. Sriprasert, N. Mitchell Chadwick

**Affiliations:** Department of Obstetrics and Gynecology, Keck School of Medicine of USC, Los Angeles, CA, United States

**Keywords:** social determinants of health (SDOH), patient-physician relationship, social needs, first-person narrative, obstetric, medical education, video

## Abstract

**Background:**

Conventional medical education lacks the lived experiences of patients which may authentically convey the social determinants of health (SDOH) and resulting health disparities. Videos of first-person patient narratives may prove a valuable education tool in this regard. The objective of this study is to investigate how patient demographics, satisfaction with care, and patient-physician relationships influence obstetric patient interest and willingness to contribute to a SDOH video curriculum by sharing their lived experiences through first-person narratives.

**Methods:**

Study design included an anonymous, cross-sectional survey and an optional semi-structured telephone interview. Participants were 18 years old with a live-birth delivery <8 weeks prior to recruitment and received care during their pregnancy at Los Angeles General Medical Center (LAGMC). Variables surveyed included demographics, satisfaction with care, aspects of the patient-physician relationship, perceived utility, and personal interest in contributing to an educational SDOH video. A bivariate analysis was conducted to compare participants’ characteristics and responses on interest in contributing and perceived helpfulness of first-person patient SDOH videos.

**Results:**

72.43% of participants (*N* = 70) believed a patient's first-person video on SDOH would be “Helpful” in preparing physicians to provide competent medical care; however, 71.43% responded “No” to “Interest” in sharing with physicians their experiences with SDOH. English preference and being U.S. born were factors significantly associated with viewing first-person SDOH video as “Helpful” (*P* > 0.001). Major themes from telephone interviews reflected enthusiasm for first-person patient narratives and perceived benefits of using patient experiences to educate physicians on SDOH. However, participants cited barriers to disclosing SDOH including brief and strictly clinical interactions with physicians, lack of continuity of care, and fear of being judged by physicians.

**Conclusion:**

While most participants recognized the utility of addressing social needs in medical education and reported satisfaction with their obstetricians and care, these factors did not uniformly translate into willingness to contribute first-person patient narratives. To improve the representation of patients from racial, ethnic, gender, linguistic, and sexual minorities into medical curricula, further research and strategies are needed to overcome the barriers discouraging patient disclosure of social needs to physicians.

## Introduction

Medical academic institutions are increasingly dedicating efforts to collaborate with patients in training both current and future physicians, with a specific focus on imparting knowledge about the social determinants of health (SDOH)—defined as the social circumstances, environment, and behavioral patterns that significantly influence health outcomes (1–4). This is reflective of the efforts to encourage physician incorporation of patient concerns and circumstances in medical management, to deliver patient-centered care (5–8). Theoretically this approach has the potential to combat health disparities experienced by groups that have been disproportionately affected, to correct physician bias, and progress toward equitable health outcomes for diverse populations (9). However, systematic review of SDOH and patient-driven curricula reveal a notable inconsistency and several barriers to incorporating the diverse patient perspectives needed to achieve this goal (10, 11).

The underrepresentation of historically marginalized groups in medical academia and the health disparities they face have profound origins in systemic issues of racism and discrimination (12–15). Within the field of Obstetrics and Gynecology, much research exists demonstrating that persons of color are at higher risk for severe maternal morbidity and mortality (16). These findings prompt a call to action to address the SDOH influencing these glaring health disparities. As the medical community strives for inclusivity in patient-driven curricula, it is crucial to develop curricula that recognize these factors and inspire physician action to address these multifaceted barriers (17). Societal and individual factors pose significant hurdles for patients in sharing their unique perspectives with medical learners, including patient constraints due to their state of health, mobility, work, finances, personal commitments, health literacy and language barriers (11, 18). Concerns about privacy and fear of judgment, often rooted in historical injustices, coupled with the fear of encountering physician bias, create a formidable barrier for patients to openly share their social needs with medical learners (19–21). These factors may be compounded for groups that have been economically and socially marginalized. Failure to accommodate barriers hindering patient participation in curriculum development results in a missed opportunity to offer equitable engagement, especially for the individuals from groups disproportionately impacted by these challenges. This oversight and passive approach in curricula development permits these voices and concerns to go unheard. Thus, to ensure an accurate reflection of patient perspectives to medical learners and increase integration of underrepresented voices in medical education, efforts are needed to develop patient driven SDOH curricula that foster an environment where these patients feel heard, valued, and respected, minimize patient burden, and provide equitable opportunities for diverse patient participation (22).

We propose that short video documentaries of first-person patient narratives on socio-cultural and environmental factors impacting their health may be an effective method to improve representation of diverse patient voices in medical academia. From the perspective of the medical learner, patient narratives have already shown great benefit in improving learner empathy towards patients and augment understanding of disease through emotional association (23–25). The addition of visuals provided by video documentation of patient experiences may enrich medical learner understanding of the aspects of a patient's life which influence health outcomes (26). But, more importantly, first person narratives have shown potential as a method for capturing underrepresented voices in medicine, while prioritizing content the storytellers wish physicians to understand (27). Ideally, with a more autonomous role as physician educators, patients may challenge the stereotypes, biases, and power dynamics that previously limited their representation in educational contexts. We hypothesize that by providing a platform where patients can authentically share their stories, with visuals to demonstrate the nuances of the SDOH, this forum will deliver an accurate representation and challenges faced by diverse populations in healthcare settings.

A significant advantage of utilizing video as a media to deliver patient narratives lies in its potential to foster critical reflection among healthcare professionals and drive quality improvement in the medical system (28). The approach of developing a mini documentary or first-person patient video narrative capturing patient interactions with medical systems, socio-cultural and daily environments, aligns with the principles of video-reflexive ethnography. As institutions have begun utilizing this method for improving systems for delivering patient-centered care (29), it is reasonable to consider this method as a way for addressing medical learner preconceptions about items that can be changed within medical systems to address health disparities experienced by patients of underrepresented backgrounds. Therein also lies the potential that patients who provide their stories will have their social needs addressed in the discovery and improvement of the systemic issue by an engaged team of healthcare providers.

Thus, video patient-driven narratives on the SDOH have the potential to authentically demonstrate underrepresented voices and inspire change or safeguards against medical injustices. Theoretically, the use of a video curricula may accommodate patient societal and individual factors which pose as barriers to traditional methods of participation in medical education (e.g., limiting the need for repeated commitments to attend live medical lectures to share their narratives). However, prior to the development and assessment of this curricula, further research is needed to understand underrepresented patients’ views of and willingness to contribute first-person narrative videos to teach SDOH to medical learners and combat systemic issues of racism and discrimination in medicine.

The objectives of this study are (1) To describe participants’ responses on patient-physician relationships and their experience of physician bias toward personal attributes; (2) To conduct a comparison between participants with and without perceived utility, exploring their characteristics, responses, language preferences, and personal interest in contributing narratives, (3) To describe the qualitative responses among participants who participated in the telephone interview regarding the sharing of their experiences on video to teach medical students the importance of awareness of SDOH. Our investigative team will turn to the patient population at the Los Angeles General Medical Center (LAGMC), one of the county's main safety-net hospitals, which predominantly serves the areas of Metro and East Los Angeles: historically, under-resourced communities, burdened with socially determined poorer health outcomes (30).

## Materials and methods

### Target population

Our study aims to provide focused exploration of racial/ethnic minority obstetric patient experiences with physicians throughout the ante-, peri-, and postpartum periods, to direct the development of the proposed curriculum that will improve medical learner awareness and regarding the SDOH contributing to poorer maternal health outcomes for this patient population. As previously noted, these experiences and a patient's willingness to participate in medical education can be influenced by the patient-physician relationship or patient satisfaction with care, perceived physician competency, and overall trust in physicians. To better control for the frequency of patient-physician interactions as a potential influencing factor, a suitable sample would consist of individuals who share similar healthcare goals and engage with physicians within a comparable timeframe and at regularly scheduled intervals. Our focus was therefore directed toward the obstetric patients at LAGMC given this population's routine interactions with physicians during prenatal and postnatal care.

### Ethics review

This study was reviewed and approved by the Institutional Review Board of the University of Southern California.

### Participant recruitment

We included postpartum patients who were at least 18 years old with a live-birth delivery less than 8 weeks prior and received care during their pregnancy from physicians at LAGMC. We excluded patients who did not have at least one prenatal care visit at LAGMC prior to delivery of their most recent pregnancy. The recruitment was conducted during the postpartum admission and/or postpartum visit at the LAGMC. By reviewing the patient's chart from July 2022 to February 2023, patients who met our study population criteria were approached by co-investigators and provided an information sheet with explanation of the content, risks and benefits associated with the opportunity for voluntary completion of a 30-question survey. The anonymous electronic survey was administered using REDCap, a HIPAA compliant research electronic data capture application. Participants who agreed to participate in the study and completed the survey were compensated for their time with a $20 gift card.

### Survey content

Demographic questions focused on patient identity (gender, race/ethnicity) and obstetric outcomes (e.g., parity, gestational age of most recent pregnancy, delivery mode). In this survey, “non-medical personal challenges” was used to describe to participants the SDOH in layman's terms. Participants were invited to provide their opinions on the following topics: (1) Aspects of the patient-physician relationship shared between the participants and their obstetricians (2) Personal labor and delivery experience (3) Perceived value of conversations with physicians about non-medical personal challenges (4) Interest in providing personal narratives on non-medical personal challenges with physicians (5) Belief in utility of first-person video narratives on the effects non-medical personal challenges have on medical experiences and health outcomes, to educate physicians (6) Physician bias experienced by patients impacted by language/race/insurance type. Questions regarding the patient-physician relationship were adapted from the validated Van-der Feltz-Cornelis et al. survey (referred to as, PDRQ-9) (31), through which patients may report satisfaction with physician helpfulness, empathic understanding, interpersonal openness, availability, etc. Our survey was offered in English and Spanish. The survey was developed in English and translated into Spanish text by our bilingual investigators.

### Telephone interview

Upon the completion of the initial survey, participants were given the opportunity to participate in an optional semi-structured telephone interview within six months of completing the initial survey. Telephone interviews were designed to last 30 min, providing participants an opportunity to elaborate on their responses in the survey, and were conducted in the participants’ preferred language (English vs. Spanish). The interview guide was designed to elicit participant reasoning for answering yes/no to interest in sharing non-medical personal challenges with physicians, perceived helpfulness of a first-person patient narrative video to educate physicians on non-medical personal challenges, perceived helpfulness of having conversations on non-medical personal challenges with physicians during medical visitations, and perception of physician familiarity with patient non-medical personal challenges. Examples of non-medical personal challenges were provided to participants at the start of the interview and participants were also encouraged to reflect and share anecdotes with interviewers on their own challenges that they believed would be educational for physicians. Participants were compensated for their time with a $50 gift card following completion of the interview. Telephone interviews were recorded using the app CallRev and were transcribed and/or translated into English text by our bilingual investigators.

### Statistical analysis

Demographics of the study population and their survey responses were presented with descriptive statistics. Frequency and proportion for responses were reported. Then bivariate analysis was conducted to compare factors linked to participants’ perceived utility of first-person patient narrative video (helpful/not helpful), participants’ interest (yes/no) in contributing to the study, and participants responding to the survey in English vs. Spanish. Chi-square test was conducted with the significant level of 0.05 using STATA 16 (College Station, TX: StataCorp LP).

Qualitative analysis and coding of phone interview transcripts was conducted using the RADaR technique (32), independently by two bi-lingual researchers. Transcript codes were selected by the two researchers to reflect recurrent themes in participant reasoning for answers given in the qualitative survey. Major themes were selected to reflect codes frequently cited by both researchers.

## Results

### Survey participant demographics

A total of 90 individuals who met the inclusion criteria were approached to complete our electronic survey; 20 individuals chose not to participate in the study, achieving a response rate of 78%. The demographic information of survey participants is depicted in Table 1. Of the (*N* = 70) survey participants, 81.43% identified as Latino. The mean age of participants was 29.13 (SD 5.29). Most participants indicated their preferred language as English 71.43% while 27.14% preferred Spanish. More than half (60%) of participants were U.S. born. Approximately one-third (35.72%) of the participants are unemployed and most of the participants (87.14%) used government sponsored insurance. Regarding the delivery history, almost all participants (98.57%) had a live birth 6 days or less prior to the survey; 60% had vaginal delivery and 38.57% had a C-section. Most of the participants (81.43%) delivered at term (gestational age ≥37 weeks). Demographic information was not collected on individuals who chose not to participate in the study.

**Table 1:** Demographics of study participants.

	Frequency (%) *N* = 70
Preference for english	
Yes	50 (71.43%)
No	19 (27.14%)
No response	1 (1.43%)
Born in US	
Yes	42 (60%)
No	24 (34.29%)
No response	4 (5.71%)
Race/ethnicity	
Asian or Asian American	4 (5.71%)
Black or African American	4 (5.71%)
Latino or Spanish (Hispanic)	57 (81.43%)
Middle Eastern or North African	2 (2.86%)
White	3 (4.39%)
Employment status	
Employed (Full-time)	18 (25.71%)
Employed (Part-time)	13 (18.57%)
Employed and student	1 (1.43%)
Student, NOT employed	2 (2.86%)
Not employed and NOT student	23 (32.86%)
Other	11 (15.71%)
No response	2 (2.86%)
Health insurance	
Government sponsored insurance	61 (87.14%)
Private insurance	2 (2.86%)
Employer sponsored	1 (1.43%)
I don't know	2 (2.86%)
No insurance	2 (2.86%)
No response	2 (2.86%)
First delivery	
Yes	30 (42.86%)
No	39 (55.71%)
No response	1 (1.43%)
Days since delivery	
6 days or less	69 (98.57%)
2 weeks	1 (1.43%)
Mode of delivery	
Vaginal	40 (57.14%)
Vacuum	2 (2.86%)
Planned C-section	10 (14.29%)
Unplanned C-section	8 (11.43%)
Emergency C-section	9 (12.86%)
No response	1 (1.43%)
Infant’s gestational age	
<28	1 (1.43%)
28–32	1 (1.43%)
32–34	1 (1.43%)
34–37	9 (12.86%)
37–40	41 (58.57%)
>40	16 (22.86%)
No response	1 (1.43%)

### Patient-physician relationship and perceived bias

Across all aspects of the patient-physician relationship cited in this survey, the majority (92.86%) of participants “Strongly Agree/Agree” with being content with the treatment provided by physicians throughout their pregnancy (Table 2). 85.72% agree that their obstetric physicians allocated time to talk about non-medical personal challenges. 88.58% agree their obstetric physicians understood their non-medical person challenges affecting their health and pregnancy. 95.72% agree that their obstetric physicians provided clear explanations regarding treatment options when pregnancy related complications were encountered. 94.28% agreed with the recommendations made by their obstetric physicians. 85.72% agreed their obstetric physicians were available to provide alternative solutions when recommendations were not agreed upon. And 94.29% of patients felt their pain was appropriately managed during and after labor. Most participants reported that their race/ethnicity (78.57%), preferred language (80%), and insurance (80%) “Did not affect” the care received from their obstetric physicians.

**Table 2:** Participant responses to statements on the patient-physician relationship, perceived physician bias toward personal attributes, and labor and postpartum satisfaction.

	Frequency (%) (*N* = 70)				
Patient-physician relationship	Strongly agree	Agree	Disagree	Strongly disagree	No response
Overall I am content with the treatment provided by the doctor(s) that I worked with throughout my pregnancy	55 (78.57%)	10 (14.29%)	1 (1.43%)	3 (4.39%)	1 (1.43%)
The doctor(s) I worked with throughout my pregnancy made time to talk with me about my non-medical, personal challenges affecting my health and pregnancy.	51 (72.86%)	9 (12.86%)	0 (0%)	8 (11.43%)	2 (2.86%)
I felt that the doctor(s) that I worked with throughout my pregnancy understood my non-medical, personal challenges that were affecting my health and pregnancy.	52 (74.29%)	10 (14.29%)	1 (1.43%)	6 (8.57%)	1 (1.43%)
I think if doctors ask patients about nonmedical, personal challenges, it makes medical treatment better.	44 (62.86%)	12 (17.14%)	3 (4.39%)	9 (12.86%)	2 (2.86%)
When I had questions or when I or my baby experienced complications during labor or delivery, my doctor(s) gave me a clear explanation of what was happening and available treatment options.	57 (81.43%)	10 (14.29%)	0 (0%)	2 (2.86%)	1 (1.43%)
I agreed with the recommendations made by the doctor(s) throughout my pregnancy.	55 (78.57%)	11 (15.71%)	1 (1.43%)	2 (2.86%)	1 (1.43%)
If I could not follow through or did not agree with the recommendations made by my doctor(s) throughout my pregnancy, the doctor(s) was dedicated to helping me find a solution.	50 (71.43%)	10 (14.29%)	3 (4.39%)	5 (7.14%)	2 (2.86%)
I believe my pain was managed appropriately during and after labor.	59 (84.29%)	7 (10%)	2 (2.86%)	1 (1.43%)	1 (1.43%)
Perceived physician bias toward personal attributes	Positively affected	Negatively affected	Did not affect		No response
I believe my race/ethnicity ______ the care I received from the doctor(s) I worked with throughout my pregnancy.	14 (20%)	0 (0%)	55 (78.57%)		1 (1.43%)
I believe my preferred language ______ the care I received from the doctor(s) I worked with throughout my pregnancy.	11 (15.71%)	1 (1.43%)	56 (80%)		2 (2.86%)
I believe my financial situation/insurance coverage ______ the care I received from the doctor(s) I worked with throughout my pregnancy.	13 (18.57%)	0 (0%)	56 (80%)		1 (1.43%)
Labor and postpartum satisfaction	Very good	Good	Bad	Very bad	No response
How would you rate your labor and delivery experience?	51 (72.86%)	18 (25.71%)	0 (0%)	0 (0%)	1 (1.43%)
How would you rate your postpartum (after delivery) follow up experience?	50 (71.43%)	19 (27.14%)	0 (0%)	0 (0%)	1 (1.43%)

### Perceived utility and interest in personally contributing narratives

Most participants (72.86%) believed a patient's first-person narrative video on non-medical personal challenges would be “Helpful/Very Helpful” in preparing physicians to provide competent medical care (Table 3). Satisfaction with aspects of the patient-physician relationship, race, language, and obstetric outcomes were not associated with participant's view of the utility of patients sharing SDOH with physicians (Table 4). Participants with English as their preferred language and those who were born in the US were more likely to view a first-person patient narrative video as “Helpful/Very Helpful” (*P* < 0.001). Participants who thought that race/ethnicity did not affect medical care (*P* = 0.022) and those who thought that insurance type did not affect medical care (*P* = 0.011) were more likely to view a first-person patient narrative video as “Helpful/Very Helpful.” Most participants (71.43%) were not interested in sharing their own non-medical personal challenges with physicians. We did not find the significant association between any factors with participants’ interest in providing the first-person video recordings.

**Table 3:** Participant perceived utility of a first-person patient narrative video to educate physicians on social determinants of health (SDOH) and interest in personally contributing narratives to the development of this educational tool.

	Frequency (%) *N* = 70				
Perceived utility in personally contributing narratives	Very helpful	Somewhat helpful	Not very helpful	Not at all helpful	No response
How helpful do you think showing doctors a video of community members sharing their experiences as a patient, would be in preparing doctors to provide medical care for people like you?	34 (48.57%)	17 (24.29%)	4 (5.71%)	14 (20%)	1 (1.43%)
Interest in personally contributing narratives	Yes	No			No response
Are you interested in sharing stories with doctors about personal challenges that are or have affected your health?	19 (27.14%)	50 (71.43%)			1 (1.43%)

**Table 4:** Comparison of participants’ characteristics and their responses to the perceived utility and interest in personally contributing narratives and language preferences among 64 participants who responded to questions regarding interest in sharing their own narratives, the perceived helpfulness of social determinants of health (SDOH) narratives, and language preferences.

	Interest in sharing own SDOH (*N* = 64)			Perceived helpfulness of SDOH narratives (*N* = 64)			Language preference (*N* = 64)		
	No	Yes	*p*	No	Yes	*p*	English speaking	Spanish speaking	*p*
	46 (71.88%)	18 (28.13%)		16 (25%)	48 (75%)		48 (75%)	16 (25%)	
Participants’ characteristics									
Hispanic Latino (*n* = 69)			0.62			0.41			0.019
No	8 (16%)	4 (21.05%)		2 (11.11%)	10 (19.61%)		13 (25%)	0 (0%)	
Yes	42 (84%)	15 (78.95%)		16 (88.89%)	41 (80.39%)		39 (75%)	18 (100%)	
Total	50	19		18	51		52	18	
English preferred (*n* = 69)			0.64			<0.001			<0.001
Yes	37 (74%)	13 (68.42%)		3 (16.67%)	47 (92.16%)		49 (96.08%)	1 (5.56%)	
No	13 (26%)	6 (31.58%)		15 (83.33%)	4 (7.84%)		2 (3.92%)	17 (94.44%)	
Total	50	19		18	51		51	18	
Born in US (*n* = 66)			0.68			<0.001			<0.001
No	16 (34.04%)	8 (42.11%)		15 (83.33%)	9 (18.75%)		8 (16.67%)	16 (88.89%)	
Yes	31 (65.96%)	11 (57.89%)		3 (16.67%)	39 (81.25%)		40 (83.33%)	2 (11.11%)	
Total	47	19		18	48		48	18	
Employment status (*n* = 68)			0.16			0.38			0.37
Employed (Full-time)	15 (30.61%)	3 (15.80%)		2 (11.11%)	16 (32%)		16 (32%)	2 (11.11%)	
Employed (Part-time)	8 (16.33%)	5 (26.32%)		4 (22.22%)	9 (18%)		10 (20%)	3 (16.67%)	
Employed and student	1 (2.04%)	0 (0%)		0 (0%)	1 (2%)		1 (2%)	0 (0%)	
Student, NOT employed	0 (0%)	2 (10.54%)		1 (5.56%)	1 (2%)		1 (2%)	1 (5.56%)	
Not employed and NOT student	16 (32.65%)	7 3 (6.84%)		6 (33.33%)	17 (34%)		16 (32%)	7 (38.89%)	
Other	9 (18.37%)	2 (10.53%)		5 (27.78%)	6 (12%)		6 (12%)	5 (27.78%)	
Total	49	19		18	50		50	18	
Highest education (*n* = 68)			0.17			0.06			0.008
GED	19 (38%)	10 (55.56%)		13 (72.22%)	16 (32%)		15 (30%)	14 (77.78%)	
Some college	12 (24%)	4 (22.22%)		2 (11.11%)	14 (28%)		16 (32%)	0 (0%)	
Associates degree	4 (8%)	2 (11.11%)		0 (0%)	6 (12%)		6 (12%)	0 (0%)	
Bachelor's degree	5 (10%)	1 (5.56%)		2 (11.11%)	4 (8%)		4 (8%)	2 (11.11%)	
Graduate professional	0 (0%)	1 (5.56%)		0 (0%)	1 (2%)		1 (2%)	0 (0%)	
None of the Above	10 (20%)	0 (0%)		1 (5.56%)	9 (18%)		8 (16%)	2 (11.11%)	
Total	50	18		18	50		50	18	
Health insurance (*n* = 68)			0.35			0.12			0.12
Government	45 (91.84%)	16 (84.21%)		15 (83.33%)	46 (92%)		46 (92%)	15 (83.33%)	
Private	1 (2.04%)	1 (5.26%)		0 (0%)	2 (4%)		2 (4%)	0 (0%)	
Employer sponsored	0 (0%)	1 (5.26%)		0 (0%)	1 (2%)		1 (2%)	0 (0%)	
Unknown	1 (2.04%)	1 (5.26%)		2 (11.11%)	0 (0%)		0 (0%)	2 (11.11%)	
No insurance	2 (4.08%)	0 (0%)		1 (5.56%)	1 (2%)		1 (2%)	1 (5.56%)	
Total	49	19		18	50		50	18	
Delivery mode (*n* = 69)						0.63			0.77
Vaginal	31 (62%)	9 (47.37%)	0.05	11 (61.11%)	29 (56.86%)		29 (56.86%)	11 (61.11%)	
Vacuum	1 (2%)	1 (5.26%)		0 (0%)	2 (3.92%)		2 (3.92%)	0 (0%)	
Planned C-section	4 (8%)	6 (31.58%)		4 (22.22%)	6 (11.76%)		7 (13.73%)	3 (16.67%)	
Unplanned C-section	8 (16%)	0 (0%)		1 (5.56%)	7 (13.73%)		7 (13.73%)	1 (5.56%)	
Emergency C-section	6 (12%)	3 (15.79%)		2 (11.11%)	7 (13.73%)		6 (11.76%)	3 (16.67%)	
Total	50	19		18	51		51	18	
Weeks pregnant (*n* = 69)			0.26			0.73			0.73
<28	1 (2%)	0 (0%)		0 (0%)	1 (1.96%)		1 (1.96%)	0 (0%)	
28–32	1 (2%)	0 (0%)		0 (0%)	1 (1.96%)		1 (1.96%)	0 (0%)	
32–34	0 (0%)	1 (5.26%)		0 (0%)	1 (1.96%)		1 (1.96%)	0 (0%)	
34–37	5 (10%)	4 (21.05%)		1 (5.56%)	8 (15.69%)		8 (15.69%)	1 (5.56%)	
37–40	29 (58%)	12 (63.16%)		13 (72.22%)	28 (54.9%)		28 (54.9%)	13 (72.22%)	
>40	14 (28%)	2 (10.53%)		4 (22.22%)	12 (23.53%)		12 (23.53%)	4 (22.22%)	
Total	50	19		18	51		51	18	
First delivery (*n* = 69)			0.49			0.31			0.12
Yes	23 (46%)	7 (36.84%)		6 (33.33%)	24 (47.06%)		25 (49.02%)	5 (27.78%)	
No	27 (54%)	12 (63.16%)		12 (66.67%)	27 (52.94%)		26 (50.98%)	13 (72.22%)	
Total	50	19		18	51		51	18	
Participants's responses									
Content with treatment (*n* = 69)			0.72			0.57			0.006
Strongly disagree	2 (4%)	1 (5.26%)		1 (5.56%)	2 (3.92%)		0 (0%)	3 (16.67%)	
Somewhat disagree	1 (2%)	0 (0%)		0 (0%)	1 (1.96%)		1 (1.96%)	0 (0%)	
Somewhat agree	6 (12%)	4 (21.05%)		1 (5.56%)	9 (17.65%)		10 (19.61%)	0 (0%)	
Strongly agree	41 (82%)	14 (73.68%)		16 (88.89%)	39 (76.47%)		40 (78.43%)	15 (83.33%)	
Total	50	19		18	51		51	18	
Doctor started SDOH conversation (*n* = 68)			0.62			0.94			0.74
Strongly disagree	5 (10%)	3 (16.67%)		2 (11.11%)	6 (12%)		5 (10%)	3 (16.67%)	
Somewhat disagree	0 (0%)	0 (0%)		0 (0%)	0 (0%)		0 (0%)	0 (0%)	
Somewhat agree	6 (12%)	3 (16.67%)		2 (11.11%)	7 (14%)		7 (14%)	2 (11.11%)	
Strongly agree	39 (78%)	12 (66.67%)		14 (77.78%)	37 (74%)		38 (76%)	13 (72.22%)	
Total	50	18		18	50		50	18	
Doctors understood SDOH (*n* = 69)			0.35			0.62			0.5
Strongly disagree	5 (10%)	1 (5.26%)		2 (11.11%)	4 (7.84%)		3 (5.88%)	3 (16.67%)	
Somewhat disagree	0 (0%)	1 (5.26%)		0 (0%)	1 (1.96%)		1 (1.96%)	0 (0%)	
Somewhat agree	8 (16%)	2 (10.53%)		4 (22.22%)	6 (11.76%)		8 (15.69%)	2 (11.11%)	
Strongly agree	37 (74%)	15 (78.95%)		12 (66.67%)	40 (78.43%)		39 (76.47%)	13 (72.22%)	
Total	50	19		18	51		51	18	
SDOH conversation made a difference (*n* = 68)			0.67			0.19			0.19
Strongly disagree	7 (14.29%)	2 (10.53%)		3 (16.67%)	6 (12%)		5 (10%)	4 (22.22%)	
Somewhat disagree	3 (6.12%)	0 (0%)		2 (11.11%)	1 (2%)		3 (6%)	0 (0%)	
Somewhat agree	8 (16.33%)	4 (21.05%)		1 (5.56%)	11 (22%)		11 (22%)	1 (5.56%)	
Strongly agree	31 (63.27%)	13 (68.42%)		12 (66.67%)	32 (64%)		31 (62%)	13 (72.22%)	
Total	49	19		18	50		50	18	
Answered my questions/concerns (*n* = 69)			0.67			0.68			0.68
Strongly disagree	1 (2%)	1 (5.26%)		1 (5.56%)	1 (1.96%)		1 (1.96%)	1 (5.56%)	
Somewhat disagree	0 (0%)	0 (0%)		0 (0%)	0 (0%)		0 (0%)	0 (0%)	
Somewhat agree	8 (16%)	2 (10.53%)		2 (11.11%)	8 (15.69%)		8 (15.69%)	2 (11.11%)	
Strongly agree	41 (82%)	16 (84.21%)		15 (83.33%)	42 (82.35%)		42 (82.35%)	15 (83.33%)	
Total	50	19		18	51		51	18	
Agreed with recommendations (*n* = 69)			0.7			0.72			0.139
Strongly disagree	1 (2%)	1 (5.26%)		1 (5.56%)	1 (1.96%)		1 (1.96%)	1 (5.56%)	
Somewhat disagree	1 (2%)	0 (0%)		0 (0%)	1 (1.96%)		1 (1.96%)	0 (0%)	
Somewhat agree	9 (18%)	2 (10.53%)		2 (11.11%)	9 (17.65%)		11 (21.57%)	0 (0%)	
Strongly agree	39 (78%)	16 (84.21%)		15 (83.33%)	40 (78.43%)		38 (74.51%)	17 (94.44%)	
Total	50	19		18	51		51	18	
Felt comfortable speaking up (*n* = 68)			0.51			0.57			0.12
Strongly disagree	3 (6.12%)	2 (10.53%)		2 (11.11%)	3 (6%)		3 (6%)	2 (11.11%)	
Somewhat disagree	3 (6.12%)	0 (0%)		1 (5.56%)	2 (4%)		3 (6%)	0 (0%)	
Somewhat agree	6 (12.24%)	4 (21.05%)		1 (5.56%)	9 (18%)		10 (20%)	0 (0%)	
Strongly agree	37 (75.51%)	13 (68.42%)		14 (77.78%)	36 (72%)		34 (68%)	16 (88.89%)	
Total	49	19		18	50		50	18	
Pain was managed (*n* = 69)			0.26			0.31			0.25
Strongly disagree	0 (0%)	1 (5.26%)		1 (5.56%)	0 (0%)		0 (0%)	1 (5.56%)	
Somewhat disagree	2 (4%)	0 (0%)		0 (0%)	2 (3.92%)		2 (3.92%)	0 (0%)	
Somewhat agree	6 (12%)	1 (5.26%)		2 (11.11%)	5 (9.80%)		6 (11.76%)	1 (5.56%)	
Strongly agree	42 (84%)	17 (89.47%)		15 (83.33%)	44 (86.27%)		43 (84.31%)	16 (88.89%)	
Total	50	19		18	51		51	18	
Influence of race/ethnicity (*n* = 69)			0.92			0.022			0.003
Positively affected	10 (20%)	4 (21.05%)		7 (38.89%)	7 (13.73%)		6 (11.76%)	8 (44.44%)	
Negatively affected	0 (0%)	0 (0%)		0 (0%)	0 (0%)		0 (0%)	0 (0%)	
Did not affect	40 (80%)	15 (78.95%)		11 (61.11%)	44 (86.27%)		45 (88.24%)	10 (55.56%)	
Total	50	19		18	51		51	18	
Influence of preferred language (*n* = 68)			0.67			0.06			0.06
Positively affected	7 (14.29%)	4 (21.05%)		5 (27.78%)	6 (12%)		6 (12%)	5 (27.78%)	
Negatively affected	1 (2.04%)	0 (0%)		1 (5.56%)	0 (0%)		0 (0%)	1 (5.56%)	
Did not affect	41 (83.67%)	15 (78.95%)		12 (66.67%)	44 (88%)		44 (88%)	12 (66.67%)	
Total	49	19		18	50		50	18	
Influence of finances/insurance (*n* = 69)			0.69			0.011			0.011
Positively affected	10 (20%)	3 (15.79%)		7 (38.89%)	6 (11.76%)		6 (11.76%)	7 (38.89%)	
Negatively affected	0 (0%)	0 (0%)		0 (0%)	0 (0%)		0 (0%)	0 (0%)	
Did not affect	40 (80%)	16 (84.21%)		11 (61.11%)	45 (88.24%)		45 (88.24%)	11 (61.11%)	
Total	50	19		18	51		51	18	
Labor experience (*n* = 69)			0.56			0.66			0.66
Good	14 (28%)	4 (21.05%)		4 (22.22%)	14 (27.45%)		14 (27.45%)	4 (22.22%)	
Very good	36 (72%)	15 (78.95%)		14 (77.78%)	37 (72.55%)		37 (72.55%)	14 (77.78%)	
Total	50	19		18	51		51	18	
Postpartum experience (*n* = 69)						0.98			0.52
Good	15 (30%)	4 (21.05%)	0.46	5 (27.78%)	14 (27.45%)		13 (25.49%)	6 (33.33%)	
Very good	35 (70%)	15 (78.95%)		13 (72.22%)	37 (72.55%)		38 (74.51%)	12 (66.67%)	
Total	50	19		18	51		51	18	

### Preferred survey language

Comparing between participants who responded to the survey in English vs. Spanish, the level of education attained was statistically different (*P* = 0.008), with (56.45%) English survey respondents reporting attained education higher than GED, compared to (9.5%) in Spanish survey respondents (Table 4). Spanish survey respondents were significantly more likely to be non-US born (*P* < 0.001) and were more likely to be non-content with treatment received from physicians (*P* = 0.006). Participants who responded to the survey in Spanish were more likely to report a “Positive Effect” of their race/ethnicity (*P* = 0.003) and insurance type (*P* = 0.011) on their medical care received while participants who responded to the survey in English viewed that neither had any effect.

### Phone interview participants

Of those who completed the initial survey (*N* = 70), 30 participants indicated they were interested in participating in the telephone interview with a response rate of 42.8% for interest. Contact was attempted by one of our researchers within 6 months of the interested participants completing the initial electronic survey. Of the 30 interested participants, 9 responded to recruitment and conducted a phone interview with one of our researchers with a response rate of 30% for interview participation. 7 interviews were conducted in English and 2 in Spanish. 3 of 7 English speaking participants and 2 of 2 Spanish speaking participants responded “Yes” to “Interest” in sharing their own determinants with physicians. All 9 phone interview participants reported that a first-person patient narrative video on the SDOH would be “Helpful/Very Helpful” to educate physicians on how to improve care for patients in the community. And all interviewees reported their labor and postpartum experiences were “Good/Very Good.”

### Major themes

#### A. Perceived benefit of a first-person patient narrative video: improving physician SDOH knowledge

During the phone interviews, the participants were asked to elaborate on their perceived helpfulness of first-person patient narrative video for training physicians on the social determinants of health. They recognized value in providing physicians with additional context and insight into their personal experiences affecting their health. One of the main benefits they highlighted was the exposure of physicians to real-life experiences different from their own (Table 5). Specifically, participants believed that by watching first-person patient videos, physicians would gain a deeper understanding of the diverse challenges patients face due to social determinants of health related to socioeconomic status.

**Table 5:** Major themes and thematic codes derived from participant responses in telephone interview.

Major themes	Codes	Descriptions
Barriers to sharing SDOH with physicians	Barrier_judge	Barrier to conversations about SDOH is fear of judgment
	Barrier_time	Barrier to conversations about SDOH of health is length of appointment time/physician-patient interactions
	Barrier_turnover	Barrier to conversations about SDOH is frequent physician turnover/continuity of care
Perceived benefit of a first-person patient narrative video	Diff_Experience	Having physicians learn about patient experiences different from their own
	SDOH_report	SDOH conversations improve physician report with patients (improved sense of trust)
	SDOH_relevant	SDOH conversations improve medical treatment
Aspects of the patient-physician encounter that participants desire improvement	Feedback_empathy	Patients desire more empathy from their physicians
	Feedback_communication	Patients desire more communication with their physicians

*“…If I am going through something, and I’m talking to the doctors, and I just had a baby two days ago, and I’m gonna get evicted in a day or so, because I don't have the funds, because I got a notice…The doctors don’t understand that. They pay, they rent, they have money and jobs. They don’t understand how it is to be homeless or about to be homeless. You get what I’m saying? They might understand, but they don’t relate.”*

Participants agreed that patient videos would effectively paint a vivid picture of the complex circumstances that make it challenging for patients to achieve desired health outcomes. They felt that by witnessing patients’ stories navigating the often compounding, multitude of stressors affecting patient health (e.g., financial constraints, lack of accessibility to safe housing, and medical jargon), these videos would humanize the experiences of patients and make these stressors more tangible for physicians to appreciate.

*“…People in different socioeconomic statuses are kind of oblivious to the realities and hardships that low-income community members face. And hearing it firsthand from somebody like myself who is a part of that community can be eye opening to them.”*

The participants anticipated that a better understanding of a patient’s circumstances would allow physicians to make the connection between root causes of patient illness and respective health outcomes. As the lack of context regarding patient social needs, that can only be provided by the patient, will likely steer a physician to only address superficial health complaints and symptoms.

*“…Letting the doctor know the background…to me, and my lifestyle, and what I do that is affecting my health…[The doctor] will better understand. Will help them better understand how to treat me as a patient. Sometimes they just see the surface level and don’t know the history or facts or story to the health problems.”*

Thus, only when physicians begin identifying and addressing the underlying determinants that may be exacerbating or causing their patients’ health problems, the participants believe physicians can provide relevant and realistic treatment plans tailored to address the specific challenges patients face outside of the clinical setting and ultimately work efficiently towards desired health outcomes.

*“I feel like, you know, the doctor knowing the patient better…About their personal life…would actually come to, like, some rare conclusions regarding their health. Because, like I said, a lot of things can trigger things that are affecting their health.”*

And in showing physicians patient experiences, participants reported the added benefit of teaching physicians to become comfortable with discussing social determinants, thereby creating an environment where patients feel more at ease.

*“I feel that [talking about non-medical personal challenges] helps because, when [physicians] know about our things that are not health-related, or some other things, I think they help better and it is easier for them to listen to us and pay more attention to us.”*

#### B. Barriers to sharing SDOH with physicians: short and strictly clinical interactions

Participants were also asked to elaborate on their reported satisfaction with care received during their pregnancy and encouraged to provide feedback for physicians on how to improve quality of care provided. Although all participants reported having a “good” labor or postpartum experience, many alluded to dissatisfaction with the minimal interaction and opportunity for communication between physician and patient. The main barrier repeatedly referenced by participants being the lack of time spent with a given physician due to short appointments and strictly clinical conversations (Table 5).

*“Like, I feel like they are not giving enough time with the patients. Like, doctors come like ‘Hey, what’s your problem? Let’s fix it. Okay, that’s it. I’m going to give you a prescription.’ And then they leave. If they don’t have communication skills to be able to hear the patient more, then they are just diagnosing a patient based on the findings of whatever problem they got.”*

Other participants referenced physician disinterest in patient’s concerns not related to clinical signs and symptoms, discouraging patients from engaging in meaningful conversations with physicians all together.

*“Because there were certain experiences where I was just brushed off. Like what I was feeling wasn’t important to them. While I felt like it was important.”*

An additional factor experienced by patients receiving care at LAGMC barring patient-physician communication is the frequent changes in designated physicians due to the nature of the safety-net and teaching hospital system. This continuous turnover poses a barrier for patients to establish a sense of comfort and trust necessary for open discussions about their social determinants of health.

*“Switching doctors from doctors, you don’t get comfortable with a certain one and then you don’t feel comfortable telling them certain things.”*

The lack of continuity with physicians makes it challenging for patients to build rapport, hindering their willingness to share personal information and seek support for the social factors affecting their health. While participants acknowledge that they cannot change the system and the resulting constant change of physicians, they believe that if physicians were to engage in discussions beyond a patient’s chief medical complaint, it would provide patients with a sense that their needs are being addressed and that the physician is trying to understand and support them.

*“I get that you can’t always see the same doctor, but maybe spending a few moments during the visit. ‘Cus most of the time it is very rushed. So just taking a little extra time or a few more minutes to spend with the patients. And not disregard that or what things arise.”*

#### C. Barriers to sharing SDOH with physicians: patient discomfort and anticipated physician bias

Participants who reported “No” interest in sharing stories on their non-medical personal challenges, or social determinants of health with physicians were asked to elaborate on the reason for their disinterest. The most frequently reported contributing factor was a lack of comfort with their physicians and fear of judgment (Table 5).

*“Just ‘cus some of them are a little bit personal and I know some people will be uncomfortable sharing certain stories with people.”*

Some participants referred to prior experiences where, after becoming vulnerable and sharing intimate details with their physicians about their personal lives, the lack of physician empathy and engagement in social need resolution discouraged them from offering this information to physicians again in the future.

*“Sometimes I think that there are some doctors that do have personal opinions, I guess you could say, yeah. And I feel like since they have their own personal opinions, they don't feel like going on with the situation or the conversation that they are in.”*

*“We can talk about it all day long, but after I talk my heart out. Then what? I’m still…my problems still remain.”*

And one participant reported fear that their medical treatment may be negatively affected as knowledge of her personal circumstances would awaken physician bias.

*“Maybe something political might come up. Then giving staff and doctors the opportunity to treat me as a patient with prejudice. Or be biased based on something I might say or the way that I live my life. So that is a fear.”*

## Discussion

### Perceived utility of medical education SDOH video of first-person patient narratives

The results of our survey and focus groups reflect participant enthusiasm for the development of first-person narrative videos to inform physicians about SDOH. The benefits participants foresaw in providing context behind health problems lay in the ability to elicit physician understanding of patient social needs and inspire comprehensive medical care to address health determinants of patients facing similar challenges. However, while most participants recognized the utility of addressing social needs in medical education, this recognition did not uniformly translate into interest in contributing narratives to the development of this tool. The concerns voiced by our focus group participants that were unwilling to share their narratives revolved around barriers of rushed and strictly clinical interactions with physicians, lack of continuity of care, and fear of being judged by physicians. Patient's unwillingness to share personal aspects of their lives given their perception of judgment from physicians is not without reason. Numerous studies have demonstrated physicians are not immune to implicit bias. This bias leads to the application of stereotypes to specific populations, such as minorities or obese patients, influencing clinical decisions and detrimentally impacting health outcomes (33–36). Interestingly, although most of our participants agreed that their experience with physicians reflected the aspects of a strong patient-physician relationship and were satisfied with the treatment received throughout their most recent pregnancy, it appears these factors were not enough to motivate participants and outweigh their concerns in sharing their narratives with physicians.

Participant discomfort regarding social need conversation seems to carry greater weight than the influence exerted by the patient-physician relationship. These results echo findings from prior investigations focused on patient social need screening, wherein it was discovered that patients become increasingly uneasy about disclosing sensitive information as the number of social needs they face accumulates (37). It is crucial to highlight that our study's participant population reflects the demographics of the Los Angeles County safety net hospital—a population at high risk for health burdens and social needs, predominantly Hispanic, utilizing government assistance insurance programs, with lower levels of education and socioeconomic status. Our findings support our understanding that an exploration of methods to encourage patients to comfortably share their experiences with SDOH, is crucial to improvement in representing individuals from these demographics in patient driven SDOH medical curricula.

Notably, prior to asking participants to contribute their narratives, the aspects of anonymity were not explicitly addressed with participants and assurance regarding the confidentiality of their personal information presented in the proposed videos was not provided. As a result, a pertinent question arises of whether patients would be more inclined to share their narratives if given the option of having their stories portrayed without disclosing the speaker’s identity. However, it is important to be aware that specific populations harbor justified distrust towards the medical system due to a history of prolonged mistreatment. Hence, a crucial component of incorporating these perspectives involves building trust and offering patients a platform to share their experiences without concerns about judgment or exploitation (38). Moreover, this consideration underscores the utmost importance of addressing ethical concerns and ensuring patient safety when implementing innovative approaches in medical education.

A compelling direction for future research could involve investigating the safeguards patients would prefer to increase their willingness to contribute narratives. Furthermore, a more in-depth exploration of the motivations and the interplay between types, quantity, and nature of social needs and willingness to contribute personal narratives is necessary to align with patient preferences and develop a curriculum that they find useful. The valuable insights gleaned from such research could significantly inform curriculum developers, facilitating the bridging of representation gaps and the enhancement of diversity in medical education.

A strength of this study lies in the successful inclusion of participants identifying as racial and ethnic minorities, which are often underrepresented in both medical research and investigations related to social determinants of health. This methodology shed light on potential variations among individuals within the same ethnic group, highlighting the influence of cultural elements and educational backgrounds on their levels of engagement with medical education and perceived utility of social needs programming. The Hispanic population encompasses a significant degree of diversity, a characteristic that is also reflected among our study participants. Notably, those who completed the English survey were born in the United States and possessed higher levels of education, in contrast to the Spanish survey participants who were more likely to be non-U.S. born. Considering the significantly higher perceived helpfulness by English survey respondents, it is conceivable that a heightened awareness of the impact of social needs on health status could serve as a motivating factor for these individuals to support the use of patient social need information in medical care (39). Our results also suggest there may be potential differences in perspectives regarding racism and physician bias seen amongst Hispanic participants. While our participants were predominantly Hispanic, Spanish survey respondents demonstrated notably higher levels of dissatisfaction with the obstetric care. However, Spanish survey respondents were more likely to report their race/ethnicity and insurance acted as protective factors on the quality of medical treatment received. These findings suggest that care expectations and perceptions of physician bias may vary amongst patients of the same race/ethnicity with different social backgrounds.

### Limitations

Limitations of this study include a small sample size and lack of generalizability. The *n* = 9 participants willing to participate in our optional telephone interview may reflect a skewed point of view and may not reflect a more generalized consensus when it comes to willingness to share stories. In addition, we did not record the demographic information or motivations of patients who did not wish to participate in our study. It is possible that patients who have had impactful experiences or strong opinions regarding the quality of their care may be more inclined to participate in sharing their experiences. Additionally, this population represents a community reflective of Los Angeles County, but these opinions may not be generalizable to other settings or populations.

When addressing the use of video media to share patient’s own personal narratives, the decision was made to not provide further information or examples of the video format presentation. This was done to limit participant bias toward the use of video as a media to include patient perspectives in medical education. This is a limitation in our study as interest in participation may differ if participants were provided with an example video in which they could assess how confidentiality would be maintained and how they would remain in control of their narrative, as a common barrier to participation noted by participants was concern for privacy.

To address potential differences in frequency of physician-patient interactions amongst participants, this study employed a specific participant demographic that required recurrent engagement with physicians through scheduled antepartum care. However, despite this approach, participants from our focus group still voiced experiences of encountering multiple physicians over the course of their pregnancies. This frequent rotation of physicians was identified as a notable barrier to establishing the level of comfort necessary to engage in candid conversations about non-medical personal challenges. This is a limitation in our study's design, as we did not record the exact number of physicians each participant interacted with. This absence of data prevents us from drawing specific correlations between the frequency of physician changes and patient satisfaction. Furthermore, the discussions conducted in our focus groups revealed that nursing staff also played a significant role in patient reflection of care received. Consequently, attributing patient satisfaction solely to interactions with physicians would be an oversimplification of the complex dynamics within a healthcare setting. However, in selecting our sample population, our study's findings also highlight the constraints posed by the teaching hospital environment, particularly in terms of continuity of care. Continuity of care, a fundamental aspect of healthcare, is essential not only for patient well-being but also for medical learners to effectively screen for and address patients’ needs. The challenges associated with maintaining this continuity can impact the rapport between patients and physicians, potentially hindering the ability to initiate sensitive conversations about social determinants of health. Another study limitation was not explicitly inquiring about participants’ individual social needs to ensure survey brevity.

## Data Availability

The raw data supporting the conclusions of this article will be made available by the authors, without undue reservation.
